# *Cleistocalyx nervosum* var. *paniala* Berry Seed Protects against TNF-α-Stimulated Neuroinflammation by Inducing HO-1 and Suppressing NF-κB Mechanism in BV-2 Microglial Cells

**DOI:** 10.3390/molecules28073057

**Published:** 2023-03-29

**Authors:** Sakawrat Janpaijit, Chanin Sillapachaiyaporn, Atsadang Theerasri, Somsri Charoenkiatkul, Monruedee Sukprasansap, Tewin Tencomnao

**Affiliations:** 1 Clinical Biochemistry and Molecular Medicine, Department of Clinical Chemistry, Faculty of Allied Health Sciences, Chulalongkorn University, Bangkok 10330, Thailand; 2 Department of Clinical Chemistry, Faculty of Allied Health Sciences, Chulalongkorn University, Bangkok 10330, Thailand; 3 Institute of Nutrition, Salaya Campus, Mahidol University, Nakhonpathom 73170, Thailand; 4 Food Toxicology Unit, Institute of Nutrition, Salaya Campus, Mahidol University, Nakhonpathom 73170, Thailand; 5 Natural Products for Neuroprotection and Anti-Ageing Research Unit, Chulalongkorn University, Bangkok 10330, Thailand

**Keywords:** *Cleistocalyx nervosum* var. *paniala*, MAPKs, microglial cells, neuroinflammation, NF-κB, TNF-α

## Abstract

Sustained inflammatory responses have been implicated in various neurodegenerative diseases (NDDs). *Cleistocalyx nervosum* var. *paniala* (CN), an indigenous berry, has been reported to exhibit several health-beneficial properties. However, investigation of CN seeds is still limited. The objective of this study was to evaluate the protective effects of ethanolic seed extract (CNSE) and mechanisms in BV-2 mouse microglial cells using an inflammatory stimulus, TNF-α. Using LC-MS, ferulic acid, aurentiacin, brassitin, ellagic acid, and alpinetin were found in CNSE. Firstly, we examined molecular docking to elucidate its bioactive components on inflammation-related mechanisms. The results revealed that alpinetin, aurentiacin, and ellagic acid inhibited the NF-κB activation and iNOS function, while alpinetin and aurentiacin only suppressed the COX-2 function. Our cell-based investigation exhibited that cells pretreated with CNSE (5, 10, and 25 μg/mL) reduced the number of spindle cells, which was highly observed in TNF-α treatment (10 ng/mL). CNSE also obstructed TNF-α, IL-1β, and IL-6 mRNA levels and repressed the TNF-α and IL-6 releases in a culture medium of BV-2 cells. Remarkably, CNSE decreased the phosphorylated forms of ERK, p38MAPK, p65, and IκB-α related to the inhibition of NF-κB binding activity. CNSE obviously induced HO-1 protein expression. Our findings suggest that CNSE offers good potential for preventing inflammatory-related NDDs.

## 1. Introduction

Microglia are the resident immune cells ubiquitously distributed in the brain, which play a fundamental role in inflammatory processes by removing various pathogens and cellular debris in the central nervous system (CNS) [1]. However, chronic microglial activation (neuroinflammation) results in the overproduction of inflammatory molecules, including tumor necrosis factor-α (TNF-α), interleukin 1β (IL-1β), interleukin 6 (IL-6), nitric oxide (NO), and reactive oxygen species (ROS) [2,3]. Increases in levels of NO are controlled by the activity of inducible nitric oxide synthase (iNOS) [4] and this enzyme is inhibited by the enhancement of heme oxygenase 1 (HO-1), an enzyme involved in oxidative and inflammatory processes [5,6]. The inhibition of the excessive inflammatory mediators could attenuate the consequences of inflammatory-mediated neuronal degeneration, which is commonly observed in various neurodegenerative disorders, including Parkinson’s disease (PD), Alzheimer’s disease (AD), Huntington’s disease (HD), multiple sclerosis (MS), and amyotrophic lateral sclerosis (ALS) [7].

TNF-α, a potent mediator of the inflammatory response, is mainly generated from the transmembrane TNF-α to be soluble TNF-α and released from the glial and neuronal cells to induce several downstream signaling cascades via binding to two types of receptors, tumor necrosis factor-α receptor 1 (TNFR1, p55) and tumor necrosis factor-α receptor 2 (TNFR2, p75). Signaling through TNFR1 triggers the immune response and apoptosis, whereas TNFR2 is responsible for cellular protection and anti-inflammation [8]. TNF-α/TNFR1 signaling cascades are initiated by the activation of mitogen-activated protein kinases (MAPKs), which include the c-Jun N-terminal kinases (JNKs), the extracellular signal-regulated kinases (ERKs) and the p38MAPKs, and the nuclear factor kappa-light-chain-enhancer of activated B cells (NF-κB). These two signaling pathways regulate several biological functions such as immune responses and cell death in the CNS [9]. Under pathological conditions, TNF-α modulates the acute inflammatory response to protect against brain injury and repair tissue damage. In contrast, the excessive activation and dysregulation of TNF-α promote persistent inflammation. Several lines of evidence reported that these effects are involved in multiple inflammatory-related diseases and neurodegeneration [10,11].

Non-steroidal anti-inflammatory drugs (NSAIDs), which act as cyclooxygenases (COX) inhibitors, are traditionally used to treat many inflammatory-mediated diseases; however, they provide undesirable side consequences to various organs such as the liver, kidneys, and gastrointestinal tract [12]. Therefore, natural plants and their bioactive compounds have gained attention due to their anti-inflammatory effects by blocking TNF-α-mediated signaling to treat TNF-α-related inflammatory disorders. *Cleistocalyx nervosum* var. *paniala* (CN) is an edible and indigenous berry from the Myrtaceae family. This plant grows in the northern province of Thailand, and its ripe fruit tastes sweet and sour. In animal models, the pulp extracts of ripe CN have been reported for their inhibitory effects on oxidative-induced toxicity in the liver and kidney [13,14]. CN pulp extracts were also reported to inhibit neurotoxicity in neuronal cells and Caenorhabditis elegans (*C. elegans*) by reducing ROS production and cell death [14,15,16]. Furthermore, studies have shown the biological properties of crude extracts from CN seeds, which areagricultural waste from food processing products. CN seed extracts exerted their anticarcinogenic activity induced by chemical molecules in a rat model and exhibited antimicrobial activity [17,18]. In addition, the neuroprotective effects of CN seed extract were also investigated in amyloid beta protein 25–35 (Aβ25–35)-induced PC12 and Aβ-expressing *C. elegans* [19]. However, the number of studies focusing on the antineuroinflammatory effects of CN seeds is limited. Therefore, the objectives of our study were to investigate the antineuroinflammation of CN seed extract and to determine the underlying signaling cascades in response to TNF-α in BV-2 mouse microglial cells.

## 2. Results

### 2.1. Characterization of Phytochemical Components in CNSE

**Figure 1:**
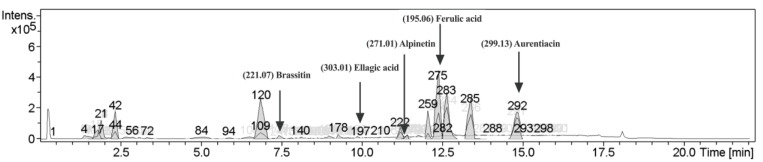
Chromatographic diagram of bioactive ingredients in ethanolic seed extract (CNSE) by liquid chromatography-mass spectrometry (LC-MS) analysis.

To determine the phytochemical profiling of CNSE, LC-MS analysis was performed, and the chromatographic peaks of CNSE in an ion-positive mode were investigated (Figure 1). Among ion peaks, five bioactive compounds were proposed to be the main components inside CNSE selected by comparing the *m*/*z* value with the MS database. These molecules included ferulic acid (40.5%), aurentiacin (30.9%), brassitin (5.4%), ellagic acid (4.5%), and alpinetin (2.0%).

### 2.2. In Silico Evaluation of Identified Compounds in CNSE against Inflammatory-Related Transcription Factors

**Figure 2:**
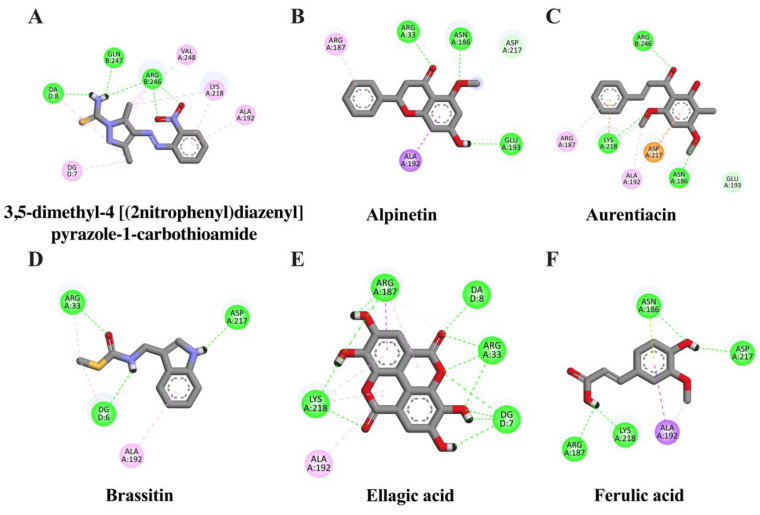
Schematic interactions of amino acid residues between reference inhibitor or candidate compounds and NF-κB protein. The original inhibitor of NF-κB protein is (**A**) 3,5-dimethyl-4-[(2-nitrophenyl)diazenyl]pyrazole-1-carbothioamide and candidate ligands are as follows: (**B**) alpinetin, (**C**) aurentiacin, (**D**) brassitin, (**E**) ellagic acid, and (**F**) ferulic acid. The green dashed line represents hydrogen, the pink or purple dashed lines represent hydrophobic bonds, and the yellow dashed line indicates other bonds.

**Figure 3:**
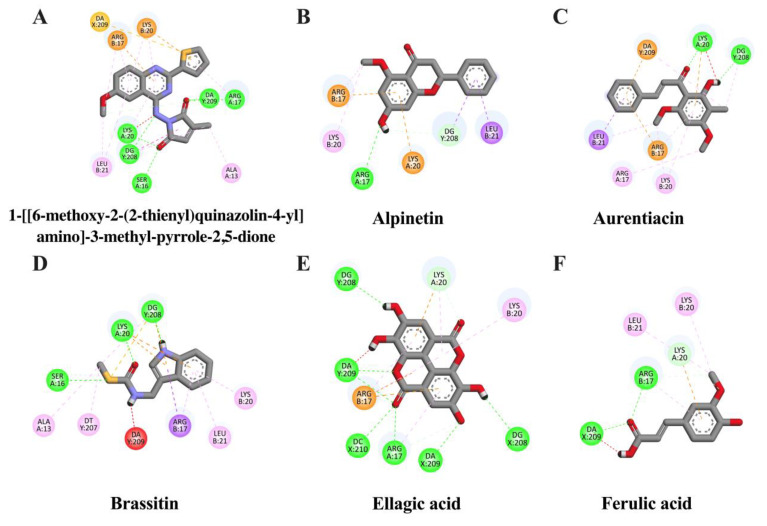
Schematic interactions of amino acid residues between reference inhibitor or candidate compounds and AP-1 protein. The original inhibitor of AP-1 protein is (**A**) 1-[[6-methoxy-2-(2-thienyl)quinazolin-4-yl]amino]-3-methyl-pyrrole-2,5-dione and candidate ligands are as follows: (**B**) alpinetin, (**C**) aurentiacin, (**D**) brassitin, (**E**) ellagic acid, and (**F**) ferulic acid. The green dashed line represents hydrogen, the pink or purple dashed lines represent hydrophobic bonds, and the yellow dashed line indicates other bonds.

It is well known that NF-κB and AP-1 are important transcriptional proteins related to inflammatory processes. Thus, to screen whether CNSE might inhibit neuroinflammation via these two mechanisms, we investigated the interaction between identified compounds in CNSE and NF-κB and AP-1 using molecular docking studies, and 3,5-dimethyl-4-[(2-nitrophenyl)diazenyl]pyrazole-1-carbothioamide and 1-[[6-methoxy-2-(2-thienyl) quinazolin-4-yl]amino]-3-methyl-pyrrole-2,5-dione were used as native inhibitors for NF-κB and AP-1, respectively. The docking result showed that a native suppressor of NF-κB, which is 3,5-dimethyl-4-[(2-nitrophenyl) diazenyl] pyrazole-1-carbothioamide, had a binding energy at −6.33 kcal/mol. Among five candidate compounds of CNSE, ellagic acid displayed a lower binding affinity (−7.31 kcal/mol), compared to the native inhibitor, whereas alpinetin (−6.17 kcal/mol) and aurentiacin (−6.01 kcal/mol) showed closer binding energies when compared to the native inhibitor as shown in Table 1. However, none of the compounds had lower or closer binding energies than a native inhibitor of AP-1, as shown in Table 2. It was possible that these candidate compounds did not associate with AP-1 protein. The interactions between the ligands and active cavities of NF-κB and AP-1 are demonstrated in Figure 2 and Figure 3. These findings suggest that CNSE might relate to the inhibition of inflammatory responses via the NF-κB signal protein. Therefore, based on this data, NF-κB was then chosen for further elucidation in our cell-based approach.

### 2.3. In Silico Evaluation of Identified Compounds in CNSE against iNOS Protein

**Figure 4:**
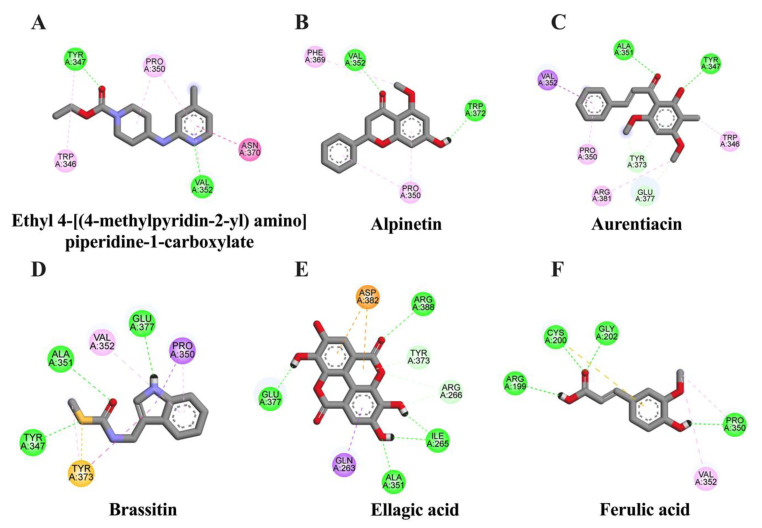
Schematic interactions of amino acid residues between reference inhibitor or candidate compounds and iNOS protein. The original inhibitor of iNOS protein is (**A**) ethyl 4-[(4-methylpyridin-2-yl) amino] piperidine-1-carboxylate and candidate ligands are as follows: (**B**) alpinetin, (**C**) aurentiacin, (**D**) brassitin, (**E**) ellagic acid, and (**F**) ferulic acid. The green dashed line represents hydrogen, the pink or purple dashed lines represent hydrophobic bonds, and the yellow dashed line indicates other bonds.

It is well studied that iNOS can be activated via NF-κB to generate a large amount of NO levels in response to inflammatory stimuli induction in glial cells [20]. To evaluate whether identified compounds in CNSE could interact with iNOS, molecular docking studies were carried out using ethyl 4-[(4-methylpyridin-2-yl) amino] piperidine-1-carboxylate [21] as an iNOS reference inhibitor. The docking result showed that ethyl 4-[(4-methylpyridin-2-yl) amino] piperidine-1-carboxylat had a binding energy at −6.91 kcal/mol and none of the compounds had lower binding energies, compared to the reference suppressor. However, among five candidate compounds, ellagic acid, aurentiacin, and alpinetin exhibited similar binding affinities to the original inhibitor at −6.32, −6.13, and −6.11 kcal/mol, respectively (Table 3) and the interactions between the ligand and active cavities of the targeted protein are demonstrated in Figure 4. These findings suggest that ellagic acid, aurentiacin, and alpinetin could inhibit inflammatory activation by blocking iNOS function.

### 2.4. In Silico Evaluation of Identified Compounds in CNSE against COX-2 Protein

**Figure 5:**
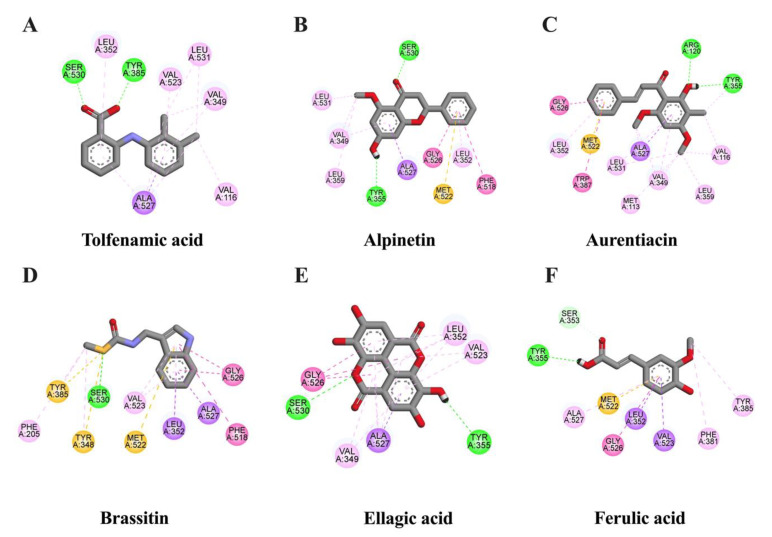
Schematic interactions of amino acid residues between reference inhibitor or candidate compounds and COX-2 protein. The reference inhibitor of COX-2 protein is (**A**) tolfenamic acid and candidate ligands are as follows: (**B**) alpinetin, (**C**) aurentiacin, (**D**) brassitin, (**E**) ellagic acid, and (**F**) ferulic acid. The green dashed line represents hydrogen, the pink or purple dashed lines represent hydrophobic bonds, and the yellow dashed line indicates other bonds.

Targeting COX-2 is one of the potential approaches to prevent the excessive production of its synthesized product, PGE2, during chronic inflammation. Molecular docking was used to predict the binding affinity between the ligands and COX-2 protein. In this study, tolfenamic acid was selected to be a native ligand for COX-2 inhibition [22], and it showed a binding score of −8.13 kcal/mol. The binding structure of candidate ligands and COX-2 is illustrated in Figure 5. As shown in Table 4, alpinetin displayed a binding energy (−8.14 kcal/mol) lower than tolfenamic acid and the docking score of aurentiacin (−8.03 kcal/mol) was also closer to the reference inhibitor. In contrast, ellagic acid, brassitin, and ferulic acid showed binding scores higher than the original inhibitor. These data indicate that both alpinetin and aurentiacin can suppress inflammatory responses by disrupting COX-2 activation.

### 2.5. Lipinski’s Rule of Five Parameters and ADMET Properties of Bioactive Components in CNSE

Lipinski’s rule of five parameters analysis was conducted to specify the bioavailable properties of drug-likeness compounds. The criteria are divided into five factors: molecular weight less than 500 Da, hydrogen bond acceptors less than ten, hydrogen bond donors less than five, MlogP less than 4.15, and compounds that showed less than one Lipinski violation were regarded for drug-likeness substances [23]. The results demonstrated that all identified compounds were approved under the criteria of Lipinski’s regulation (Table 5). Furthermore, the pharmacokinetic and pharmacodynamic properties, including the absorption, distribution, metabolism, excretion, and toxicity (ADMET) of the candidate ligands in CNSE, were investigated and are shown in Table 6. The results showed that all bioactive compounds (alpinetin, aurentiacin, brassitin, ellagic acid, and ferulic acid) provided high GI absorption with non-carcinogenicity and could penetrate the blood–brain barrier (BBB), except ellagic acid, which exerted a negative effect on BBB permeability.

### 2.6. Effect of CNSE on the Viability of BV-2 Cells

**Figure 6:**
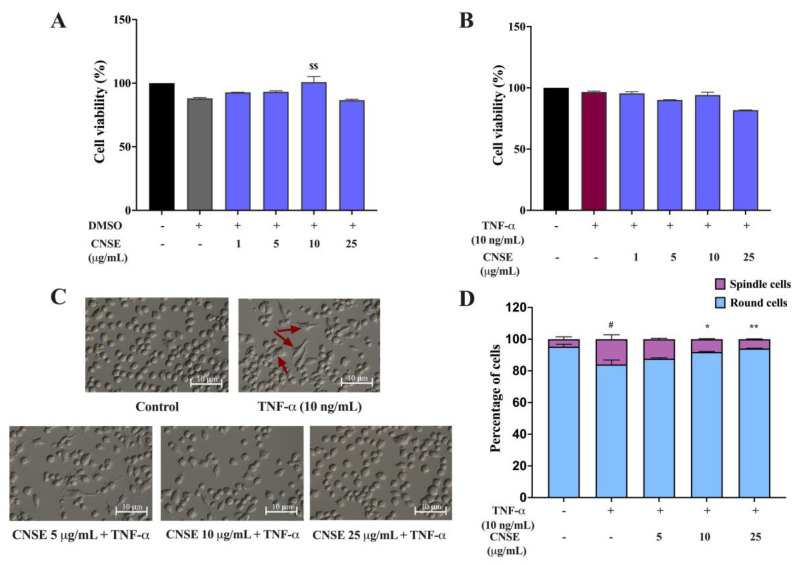
Effects of CNSE on cell viability and morphological phenotypes in BV-2 cells. Cell viability was determined using an MTT assay. For cytotoxicity assay, (**A**) BV-2 cells were incubated with various doses of CNSE for 24 h. Black color represents untreated control, grey color represents DMSO control and violet color represents CNSE treatment. (**B**) CNSE was pretreated for 24 h, followed by TNF-α for another 24 h. Black color represents untreated control, red color represents TNF-α group and violet color represents CNSE treatment. For morphological analysis. (**C**) Morphological phenotypes were captured using 10× magnification of phase-contrast microscopy after pretreatment of cells with CNSE and TNF-α for 24 h. The red arrows represent the elongated cells. (**D**) The percentage of round and spindle cells is shown as a bar graph. Data are represented as the mean ± SD from at least three independent experiments. A *p*-value < 0.05 was considered to show a significant difference between each group (^$$^ *p* < 0.01 vs. DMSO control; ^#^ *p* < 0.05 vs. untreated control; ** *p* < 0.01; * *p* < 0.05 vs. TNF-𝛼-treated group).

For the cell-based investigation, we first examined the cytotoxicity of CNSE on BV-2 cells. An MTT assay was assessed after the treatment of cells with various doses of CNSE at 0–25 μg/mL in the presence or absence of TNF-𝛼 (10 ng/mL) for 24 h. The results showed no reduction in cell viability lower than 80% in BV-2 cells upon treatment with CNSE, compared to the DMSO control (Figure 6A). In the presence of TNF-𝛼, CNSE at 25 μg/mL showed a slight reduction in cell viability compared to TNF-𝛼 treatment (Figure 6B). Therefore, CNSEs at 5, 10, and 25 μg/mL were selected to further evaluate their antineuroinflammation in TNF-𝛼-induced BV-2 cells in the next experiments.

### 2.7. Effect of CNSE on the Morphological Changes of BV-2 Cells

The morphological phenotypes of BV-2 after treatment with TNF-𝛼 were observed under a phase-contrast microscope. In the normal condition, BV-2 cells displayed numerous round and short cell bodies, whereas TNF-𝛼 could induce the morphological inversion from round to bipolar and multipolar branched cells with a cell soma enlargement, compared to the control group. After the treatment of cells with the extract, BV-2 cells showed a similar characterization as seen in control cells. The percentage of spindle cells was lowered concentration-dependently compared to the TNF-𝛼 group (Figure 6C,D). These results suggest that CNSE can prevent the morphological changes induced by TNF-𝛼.

### 2.8. Inhibitory Effect of CNSE on Levels of Proinflammatory Cytokines

**Figure 7:**
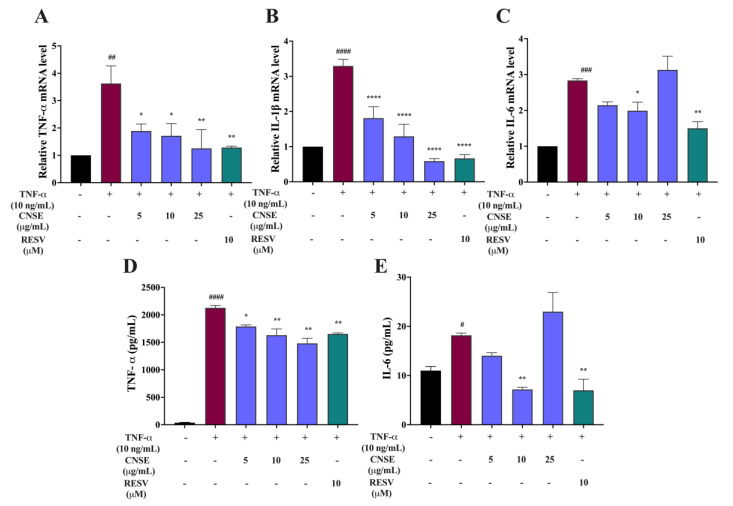
Inhibitory effects of CNSE on the expressions of proinflammatory cytokines’ production in BV-2 cells. The mRNA levels of inflammatory cytokines were measured after treatment of cells with CNSE for 24 h, followed by TNF-α for another 3 h using real-time PCR. RESV was used as a positive control. The relative expression, as shown by fold changes in (**A**) TNF-𝛼, (**B**) IL-1β, and (**C**) IL-6, was normalized with the internal control, β-actin. The release of cytokines, including (**D**) TNF-𝛼 and (**E**) IL-6, was also measured using commercial ELISA kits. The concentrations of protein levels (pg/mL) in the cell culture medium after CNSE and TNF-𝛼 treatment for 24 h were calculated by comparing them to the standard curve. Black color represents untreated control, red color represents TNF-α group, violet color represents CNSE treatment and green color represent RESV treatment. Data are represented as the mean ± SD from at least three independent experiments. A *p*-value < 0.05 was considered to show a significant difference between each group (^####^ *p* < 0.0001; ^###^ *p* < 0.001; ^##^ *p* < 0.01; ^#^ *p* < 0.05 vs. untreated control; **** *p* < 0.0001; ** *p* < 0.01; * *p* < 0.05 vs. TNF-𝛼-treated group).

Increasing levels of major proinflammatory cytokines, including TNF-α, IL-1β, and IL-6, are associated with several inflammatory-related neurodegenerative diseases [24]. To measure the gene expression levels of proinflammatory mediators, real-time PCR was carried out after the treatment of cells with CNSE in combination with TNF-α for 3 h. Resveratrol (RESV) was used as a positive control. The results showed that TNF-α enhanced the levels of TNF-α, IL-1β, and IL-6 mRNA expression, compared to the control, whereas the mRNA expression of all cytokines was suppressed after CNSE treatment in a concentration-dependent manner, compared to the TNF-α group, and 10 μM of RESV also suppressed the mRNA expression of these cytokines in TNF-α-induced BV-2 cells (Figure 7A–C). In addition, the releases of TNF-α and IL-6 in BV-2 cells were also detected using a commercial ELISA kit. After 24 h of TNF-α induction, TNF-α and IL-6 were significantly increased, compared to the untreated control. However, treatment with CNSE and RESV could inhibit the production of TNF-α (Figure 7D) and IL-6 (Figure 7E). Interestingly, 25 μg/mL of CNSE did not reduce the expression of both mRNA and protein levels of IL-6. These data suggest that CNSE has an inhibitory action on the generation of proinflammatory mediators.

### 2.9. Effect of CNSE on MAPKs Signaling Activation

**Figure 8:**
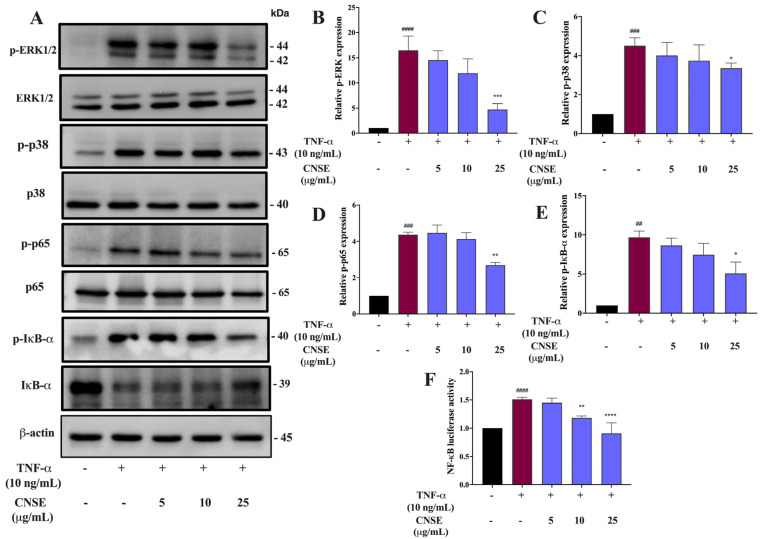
Inhibitory effects of CNSE on MAPKs and NF-κB activation. Cells were pretreated with CNSE in response to TNF-α for 5 min, and then (**A**) the protein levels of MAPKs and NF-κB were evaluated by Western blot analysis. The relative proteins levels of (**B**) phosphorylated ERK (p-ERK), (**C**) phosphorylated p38MAPK (p−p38), (**D**) phosphorylated p65 (p−65), and (**E**) phosphorylated IκB-α (p-IκB-α) are presented in the histogram graph, and each protein was quantified and normalized with β-actin. NF-κB (p−65) binding activity after treatment cells with the extract, followed by TNF-α, was investigated by performing a dual-luciferase assay. (**F**) The relative level of p65 binding activity was normalized through the activity of pRL-null and was expressed as fold changes. Black color represents untreated control, red color represents TNF-α group and violet color represents CNSE treatment. Data are represented as the mean ± SD from at least three independent experiments. A *p*-value < 0.05 was considered to show a significant difference between each group (^####^ *p* < 0.0001; ^###^ *p* < 0.001; ^##^ *p* < 0.01 vs. untreated control; **** *p* < 0.0001; *** *p* < 0.001; ** *p* < 0.01; * *p* < 0.05 vs. TNF-𝛼-treated group).

It is well established that the MAPK pathway contributes to glial-related neuroinflammation by regulating the expression of inflammatory molecules via transcription factors including NF-κB [25]. In this study, the protein level of ERK1/2 and p38MAPK was determined by Western blot analysis. Cells were pretreated with CNSE and then incubated with TNF-α for 5 min. Immunoblotting results revealed that the phosphorylated forms of both ERK1/2 and p38MAPK were induced by TNF-α, compared to the untreated group. After the pretreatment of cells with CNSE, the levels of these proteins tended to decrease when compared with the TNF-α group. Moreover, CNSE treatment at the highest dose (25 µg/mL) distinctly attenuated the level of these phosphorylated MAPK proteins, compared to the TNF-α group (Figure 8B,C). The original blots were shown in the supplement material (Figure S1). The results suggest that the antineuroinflammatory effects of CNSE are controlled by the ERK and p38MAPK mechanisms.

### 2.10. Effect of CNSE on NF-κB Signaling Activation

The transcription factor NF-κB is the major regulatory protein involved in the inflammatory process by directly promoting the production of numerous inflammatory mediators [26]. To examine whether the antineuroinflammatory roles of CNSE are involved in the NF-κB signaling pathway, a luciferase assay was performed to determine the DNA binding activity of NF-κB. The results showed that TNF-α promoted NF-κB binding activity, compared to the control, whereas CNSE suppressed TNF-α-mediated NF-κB promoter activation (Figure 8F). Thus, to confirm the inhibitory effects of CNSE on the NF-κB pathway, the protein level of phosphorylated form p65 and IκB-α was measured by performing Western blot analysis after the treatment of cells with CNSE, followed by TNF-α for 5 min. The results showed that both p65 and IκB-α were highly phosphorylated by TNF-α treatment and these effects were strongly inhibited by CNSE pretreatment, compared to the TNF-α group (Figure 8D,E). The original blots were shown in the supplementary material (Figure S1). These data indicate that CNSE has a suppressive role in the NF-κB signaling pathway, inhibiting inflammatory key proteins in BV-2 cells.

### 2.11. Effect of CNSE on HO-1 Activation

**Figure 9:**
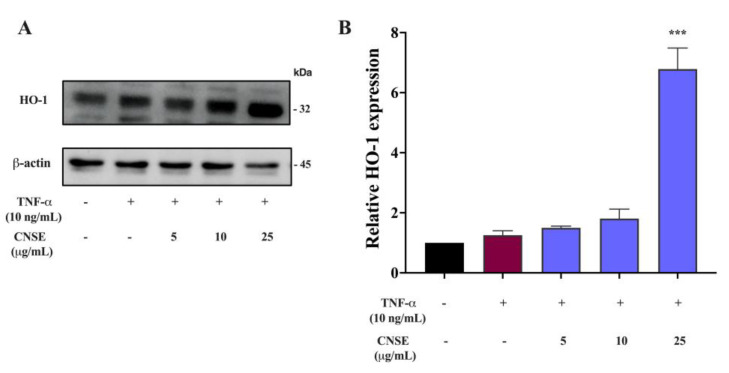
Effects of CNSE on HO-1 induction. BV-2 cells were preincubated with CNSE and then stimulated with TNF-α for 24 h. (**A**) The protein expression of HO-1 using Western blot analysis. (**B**) The histogram graph presents the relative expression of HO-1, which was quantified and normalized with β-actin. Black color represents untreated control, red color represents TNF-α group and violet color represents CNSE treatment. Data are represented as the mean ± SD from at least three independent experiments. A *p*-value < 0.05 was considered to show a significant difference between each group (*** *p* < 0.001 vs. TNF-𝛼-treated group).

We also focused on the protein expression of HO-1, which has cytoprotective enzyme and antioxidant properties, responding to inflammatory stimuli. Our results showed that CNSE treatment, especially at the highest concentration (25 μg/mL), greatly promoted the induction of HO-1 in our neuroinflammatory model of BV-2 cells, as shown in Figure 9. The original blots were shown in the supplementary material (Figure S2).

## 3. Discussion

Microglial-mediated neuroinflammation is a causative factor mainly found to be involved in numerous inflammatory-related disorders in the brain. Typically, microglia are characterized by a ramified shape and are responsible for surveying the brain to maintain synaptic and neuronal development and homeostasis. In contrast, microglial cells, represented by an amoeboid appearance, regulate the immune responses by phagocytosis and chemotaxis to eliminate any toxic substances harmful to the CNS [1,27]. The neuroprotective function of microglia can be switched to a neurotoxic effect resulting from sustained microglial activation. They are defined by an increased generation of inflammatory mediators, including TNF-α, IL-1β, IL-6, NO, prostaglandin (PGE), and ROS. Large amounts of these mediators are secreted from glial cells and cause detrimental effects on surrounding neuronal cells, eventually leading to neuronal cell damage and death [2,28].

TNF-α, a main proinflammatory protein, consists of two active forms, soluble and transmembrane proteins, which are differently responsible for various biological activities depending on their receptors: TNFR1 and TNFR2. TNF-α/TNFR1-mediated signaling cascades are involved in neurotoxic, inflammatory conditions and apoptosis, whereas TNF-α/TNFR2 signaling is associated with neuroprotective and anti-inflammatory features [29,30]. However, dysregulated TNF-α and related signaling cascades have been reported to lead to persistent increases of synaptic dysfunction, glutamatergic neurotoxicity, and chronic neuroinflammation in the brain in several neurodegenerative diseases such as AD, PS, MS, and ALS [31,32]. Previous studies demonstrated that TNF-α was used as an inflammatory inducer due to its capability to mediate inflammatory-related mechanisms in microglia cells and its direct relevance in pathological circumstances in microglial-mediated neuroinflammatory disorders [33,34]. Therefore, this study selected TNF-α to induce inflammatory characterization in BV-2 cells.

Anti-inflammatory drugs, known as NSAIDs, have been extensively used in curing inflammatory-mediated disorders by targeting the inhibition of inflammatory proteins, such as peroxisome proliferator-activated receptor-γ (PPAR-γ) and COX enzymes, COX-1, and COX-2, which are implicated in inflammatory processes. This leads to decreased PGE synthesis, which is mostly produced in microglia [35]. However, the chronic administration of NSAIDs can cause undesirable and adverse effects on various body systems, such as cardiovascular, gastrointestinal, renal, and hepatic systems [36]. Therefore, natural plants and fruits that contain several bioactive compounds have been widely studied by targeting inflammatory-related molecules and involved signaling to develop medicine for relieving the progression of neuroinflammatory-mediated diseases. This study focused on the antineuroinflammatory effects of seed extract from the *C. nervosum* var. *paniala* berry or “Ma-kiang” (local-Thai name). Several biological activities of these berry fruits have been reported, including antiaging and neuroprotective properties, anticarcinogenicity, and immunomodulation [37]. Interestingly, a part of the seed, which is an organic waste, showed high levels of polyphenols and flavonoids [38]. However, the antineuroinflammatory studies focusing on the agricultural waste part of the seed are still limited. Thus, in this study, TNF-α was used to induce inflammatory characteristics in BV-2 cells for investigating the roles of CNSE in the suppression of inflammatory-related proteins and mechanisms in response to TNF-α. Herein, CNSE possessed antineuroinflammation by inhibiting proinflammatory molecules, including TNF-α, IL-1β, and IL-6 through the regulation of NF-κB, MAPKs, and HO-1.

To identify possible bioactive components in CNSE, phytochemical profiling of CNSE was evaluated by performing LC-MS analysis. Chromatographic diagrams showed that ferulic acid, aurentiacin, brassitin, ellagic acid, and alpinetin are five major candidate ingredients of CNSE (Figure 1), which could be responsible for antineuroinflammatory properties of CNSE. Among the five compounds, ferulic acid, aurentiacin, ellagic acid, and al-pinetin have been reported to be found in natural plants and berry fruits, especially in the Myrtaceae family based on the KNApSAcK database. To screen whether CNSE might inhibit inflammatory responses via the transcription factors, namely NF-κB and AP-1, in silico screening by targeting NF-κB and AP-1 using molecular docking was performed to predict the inhibitor potential of these ligands in CNSE against targeted proteins. The docking results showed that ellagic acid had a lower binding affinity and alpinetin and aurentiacin displayed similar binding energies, compared to an NF-κB inhibitor, 3,5-dimethyl-4-[(2-nitrophenyl)diazenyl]pyrazole-1-carbothioamide (Table 1), whereas none of the compounds had lower or closer binding energies, compared to a native inhibitor of AP-1, 1-[[6-methoxy-2-(2-thienyl)quinazolin-4-yl]amino]-3-methyl-pyrrole-2,5-dione (Table 2). Three-dimensional schematics of candidate ligands, which showed amino acid residues between ligands and targeted proteins, NF-κB and AP-1, are also illustrated in Figure 2 and Figure 3. Based on this investigation, molecular docking data indicate that these five bioactive phytochemicals only linked to NF-kB but did not relate to the AP-1. Thus, in the present study, we primarily focused on investigating the effects of CNSE on NF-κB activation. However, the effects of CNSE and its bioactive compounds on AP-1 activation might need to be explored in the next cell-based assay. In addition, we subsequently determined the interaction between bioactive compounds and iNOS and COX-2, which are downstream proteins of NF-κB. The docking results demonstrated that ellagic acid, alpinetin, and aurentiacin had a similar binding affinity to the active site of iNOS, as compared to the native suppressor, ethyl 4-[(4-methylpyridin-2-yl) amino] piperidine-1-carboxylate. Both alpinetin and aurentiacin exhibited a high binding affinity to the COX-2 active pocket, as compared to the original inhibitor, tolfenamic acid (Table 3 and Table 4). Three-dimensional schematics of candidate ligands, which showed amino acid residues between ligands and targeted proteins, iNOS and COX-2, are also illustrated in Figure 4 and Figure 5. The inhibitory effects of these compounds, as investigated by docking analysis, were consistent with the previous findings. Ellagic acid has been reported for its anti-inflammatory effects in microglial cells. It has been reported that ellagic acid could inhibit the release of NO and TNF-α and the protein level of MAPKs, NF-κB, and iNOS in BV-2 cells induced by LPS [39]. In rodent-model experiments, ellagic acid treatment reduced oxidative stress and neuroinflammation via the Nrf2/ARE pathway activation and Toll-like receptor 4 (TLR4) downregulation, the upregulation of HO-1 expression and threonine-protein kinase (Akt), and the inhibition of COX-2 and 5-LOX proinflammatory signaling pathways [40]. A significant reduction in microglial activation and astrocytosis suggests the potential therapeutic benefit of ellagic acid treatment in multiple sclerosis [41]. Alpinetin, a natural dihydroflavone, has previously been shown to inhibit proinflammatory cytokines, TNF-α, IL-1β, and IL-6, via ERK/p38 and NF-κB in LPS-induced macrophages and attenuated TNF-α-induced NF-κB activation in chondrocytes [42,43]. Aurentiacin has also been demonstrated for the anti-inflammatory role in RAW264.7 macrophages by inhibiting the generation of inflammatory mediators via regulating MAPKs and NF-κB signaling cascades [44]. Other compounds, such as ferulic acid, showed a lower binding affinity binding to iNOS and COX-2 in docking results related to the anti-inflammatory properties. Several findings reported that ferulic acid could inhibit LPS-induced neuroinflammation in BV-2 cells by suppressing inflammatory proteins, including iNOS, COX-2, TNF- α, and IL-1β, and decreased inflammatory levels in a mouse model, which was induced by mild stress via the NF-κB pathway [45,46]. Our study suggests that CNSE can potentially suppress neuroinflammatory responses via modulating related signaling cascades, including MAPKs and NF-κB. Moreover, these compounds’ pharmacokinetic and pharmacodynamic properties were evaluated using ADMET analysis. The results showed that all compounds, except ellagic acid, could highly absorb in the GI tract without carcinogenic toxicity and pass through the BBB (Table 4). However, other factors, including oral bioavailability and CNS toxicity, require consideration. In cell-based experiments, the morphological phenotypes of BV-2 after the pretreatment of cells with CNSE, followed by TNF-α, were primarily observed using a phase-contrast microscope. The results showed that untreated BV-2 cells mostly expressed short, round bodies and, after TNF-α induction, the elongated cells were increased compared to the control. In contrast, CNSE treatment decreased the number of spindle cells (Figure 6C,D). In BV-2 cells, the resting state normally expressed high levels of round cells, while with inflammatory inducer treatment cells were switched to a long and multipolar morphology [47]. It is well known that, under pathological conditions, high levels of inflammatory mediators, including proinflammatory cytokines, are secreted by microglial cells to initiate inflammatory processes. Therefore, the mRNA and protein levels of major proinflammatory cytokines, including TNF-α, IL-1β, and IL-6, were examined in TNF-α-induced BV-2 cells. Our real-time PCR and ELISA results showed that TNF-α enhanced the gene expression of all cytokines and induced the release of TNF-α and IL-6 in BV-2 cells compared to the untreated group. CNSE and RESV treatment could inhibit these levels compared to the TNF-α treatment (Figure 7A–E). Interestingly, CNSE at 25 μg/mL did not significantly decrease IL-6 mRNA and protein expression. Similarly, some berry fruits, such as the goji berry, did not reduce the level of serum IL-6 in healthy subjects and increased IL-6 expression in LPS-treated rats [48,49]. Additionally, a previous study demonstrated that IL-6 could also act as an anti-inflammatory cytokine involved in neurogenesis [48]. These data collectively indicate that CNSE exerts antineuroinflammatory effects by suppressing the expression of inflammatory-related cytokines.

Based on our above finding, molecular mechanisms responsible for the suppressive effects of CNSE on neuroinflammation were further investigated. A previous study showed that TNF-α could stimulate inflammatory processes by controlling MAPKs and NF-κB mechanisms [50]. MAPKs are members of serine/threonine (Ser/Thr) protein kinases, mainly ERK, JNK, and p38MAPK. These kinases have been reported to exert cellular processes, including apoptosis, oxidative damage, differentiation, and immunological regulation. Stress and inflammatory molecules can induce the induction of MAPKs signaling pathway, which activates inflammatory processes by regulating the expression of inflammatory mediators [51]. Furthermore, it is well established that the transcription factor NF-κB is the central regulator in immune and inflammatory cascades and other physiological actions, including cell survival and proliferation, cell metabolism, behavioral action, and synaptic plasticity [52]. Under normal circumstances, NF-κB subunit protein, namely p65 or Rel A, is resident in the cytoplasm and binds to its inhibitory protein, also known as IκB-α, indicating an inactive function of NF-κB. Once induced by neurotoxic, pathogens, or cytokines, IκB-α is phosphorylated and degraded, releasing p65 into the nucleus. The translocation of p65 leads to the transcriptional activation of various inflammatory mediators for initiating inflammatory signaling cascades [53]. Therefore, targeting neuroinflammation focusing on MAPKs and NF-κB has been extensively studied in several inflammatory-mediated disorders, including neurodegenerative diseases [52,54,55]. In this study, TNF-α obviously induced the level of phosphorylated MAPKs, including ERK and p38MAPK, compared to the untreated control group. In contrast, pretreating cells with CNSE could attenuate TNF-α-induced MAPKs activation in BV-2 cells (Figure 8B,C). Numerous data showed that the antineuroinflammatory effects of the plant extracts have been reported against all three MAPK members (ERK, p38MAPK, and JNK) [20,25,49,51]. However, the effects of CNSE on JNK activation should be further investigated in the next study. In addition, TNF-α also stimulated the NF-κB activation by enhancing NF-κB promoter activity and the phosphorylated form of p65 and IκB-α, whereas CNSE also inhibited the luciferase activity of NF-κB and phosphorylated levels of p65 and IκB-α, compared to TNF-α induction (Figure 8D–F). HO-1 is the inducible enzyme responsible for converting heme to biliverdin, ferrous ion, and carbon monoxide. This enzyme has been known to inhibit the production of ROS, which in turn activates neuroinflammation, by the removal of free heme, which has pro-oxidant properties, and by the formation of biliverdin (BV) and subsequently bilirubin (BR), which have been shown to be potent antioxidants [56,57]. Additionally, HO-1 can promote the anti-inflammatory phenotype that is related to the production of anti-inflammatory cytokines and motivate the activation of interferon regulatory factor 3 (IRF3) in response to inflammatory stimuli [58]. Importantly, HO-1 is modulated via several transcription factors, including NF-κB, nuclear factor erythroid factor 2-related factor 2 (Nrf2), and activator protein 1 (AP-1), and is involved in antioxidants and neuroinflammation [59]. Previous studies reported that the induction of HO-1 is correlated with the antineuroinflammation of compounds in response to LPS in BV-2 cells [60,61]. Our result showed that TNF-α did not significantly alter the expression of HO-1. Conversely, CNSE, especially at 25 μg/mL markedly increased HO-1 protein expression (Figure 9A,B). The results suggest that CNSE exerts antineuroinflammatory effects by inhibiting inflammatory proteins and inducing HO-1 via regulating MAPKs and NF-κB.

## 4. Materials and Methods

### 4.1. Materials and Reagents

Recombinant murine TNF-α was obtained from Peprotech (Rocky Hill, NJ, USA). Dulbecco’s Modified Eagle’s Medium (DMEM), resveratrol (purity ≥ 99%), and Bradford reagent were purchased from Sigma-Aldrich Co. (St Louis, MO, USA). Fetal bovine serum, 0.25% trypsin-EDTA, and 10X Penicillin-Streptomycin were purchased from Gibco BRL (Life Technologies, Paisley, UK). 3-(4,5-dimethylthiazol-2-yl)-2,5-diphenyltetra-zolium bromide (MTT) was obtained from Bio Basic (Markham, ON, Canada). Trizol reagent was obtained from Invitrogen (Carlsbad, CA, USA). Primers, AccuPower RT-premix, and 2X GreenStarTM qPCR Master Mix were obtained from Bioneer (Daejeon, South Korea). The phosphorylated form of p65 (p-p65) (S536), p65 (D14E12), p-IκB-α (S32), IκB-α (44D4), p-ERK1/2 (D13.14.4E), ERK1/2 (137F5), p-p38 (D3F9), p38 (D13E1), and β-actin (13E5) antibodies were purchased from Cell Signaling Technology (Danvers, MA, USA). The antibody against HO-1 (A-3) was obtained from Santa Cruz Biotechnology (Dallas, TX, USA). The ELISA kit for TNF-𝛼 was obtained from Thermo Scientific (Rockford, IL, USA).

### 4.2. CNSE Extraction

CN fruits were collected from the Plant Genetic Conservation Project under the Royal Initiation of Her Royal Highness Princess Maha Chakri Sirindhorn (Lampang Province, Thailand) in July–August 2018. Asst. Prof. Dr. Thaya Jenjittikul from Department of Plant Science, Faculty of Science, Mahidol University, Bangkok, Thailand authenticated the CN. It was identified and deposited at Suan Luang Rama IX Herbarium, Bangkok, Thailand with voucher specimen No. 9428. For the preparation of CN samples, the seeds of CN fruits were separated from the pulp and ground into a powder using a food blender and lyophilized before the extraction process. Then, the extraction of lyophilized powder was carried out using a Soxhlet apparatus with 95% of ethanol as the solvent for 24 h. The sample was further evaporated by removing the solvent using a rotary evaporator at 45 to 50 °C. The yield percentage of the CN seed extract (CNSE) was approximately 6.75 % (*w*/*w*) and appeared a dark-green color. CNSE was dissolved in DMSO and filtered through 0.2 μm pore size paper as a stock solution of 100 mg/mL, which was stored in darkness at −20 °C.

### 4.3. Liquid Chromatography-Mass Spectrometry (LC-MS) Analysis

To identify the phytochemical profiling inside CNSE, the extract was sent to the Institute of Systems Biology (Universiti Kebangsaan Malaysia, Malaysia) for LC-MS analysis using a DionexTM Ultimate 3000 UHPLC system (Thermo Fisher Scientific, Rockford, IL, USA), which coupled with a high-resolution micrOTOF-Q III (Bruker Daltonics, Bremen, Germany). The chromatography was performed on an AcclaimTM Polar Advantage II C18 column (3 mm ⇥ 150 mm, 3 μm particle size) (Thermo Fisher Scientific, Rockford, IL, USA) with a mobile phase containing 0.1% formic acid in water (A) and 100% acetonitrile (B). The gradient conditions consisted of 5% B for 0–3 min; 80% B for 3–10 min; 80% B for 10–15 min; and 5% B for 15–22 min, along with 0.1% A, and a flow rate was set to 400 μL/min. Then, electrospray ionization (ESI) with an ion-positive mode was used as a detector and the *m*/*z* values were analyzed by comparing the METLIN (La Jolla, CA, USA) and the KNApSAcK (Keyword Search Web Version 1.000.01) databases, with an accepted error of molecular weight less than 30 parts-per-million (ppm).

### 4.4. Molecular Docking

#### 4.4.1. Protein Preparation

Protein structures of NF-κB (PDB ID: 2RAM, https://www.rcsb.org/structure/2RAM (accessed on 25 February 2023)) [62], AP-1 (PDB ID: 2H7H, https://www.rcsb.org/structure/2H7H (accessed on 17 February 2023)) [63], COX-2 (PDB ID: 5IKT, https://www.rcsb.org/structure/5IKT (accessed on 10 December 2022)) [64], and iNOS (PDB ID: 3E7G, https://www.rcsb.org/structure/3E7G (accessed on 10 December 2022)) [65] were obtained from the RCSB Protein Data Bank. The protein structures were prepared following the previous procedure [66]. Briefly, all water molecules and ligands were removed from the structure, then the missing hydrogens and Kollman charges were applied in the protein structures using AutoDockTools-1.5.6 software. The prepared protein structures were saved in PDBQT format for further molecular docking study.

#### 4.4.2. Ligand Preparation

All ligand structures were retrieved from the PubChem online database (https://pubchem.ncbi.nlm.nih.gov (accessed on 10 December 2022)) in SDF format. The ligand structures were minimized energy and saved in PDB format using BIOVIA Discovery Studio 2020. The PDB files were converted to PDBQT files by the AutoDockTools-1.5.6 program.

#### 4.4.3. Method Validation

To optimize the parameters used in this study, 3,5-dimethyl-4-[(2-nitrophenyl)diazenyl]pyrazole-1-carbothioamide; 1-[[6-methoxy-2-(2-thienyl)quinazolin-4-yl]amino]-3-methyl-pyrrole-2,5-dione; tolfenamic acid; and ethyl 4-[(4-methylpyridin-2-yl) amino] piperidine-1-carboxylate, the native ligands of NF-κB, AP-1, COX-2, and iNOS, respectively, were re-docked into the original binding sites of their target. The root-mean-square deviation (RMSD) of re-docking and co-crystal conformations were considered. The docking parameters that provided RMSD values lower than or equal to two angstroms [22] were used for the molecular docking study of the candidate ligands. Herein, the re-docking result of 3,5-dimethyl-4-[(2-nitrophenyl)diazenyl]pyrazole-1-carbothioamide and 1-[[6-methoxy-2-(2-thienyl)quinazolin-4-yl]amino]-3-methyl-pyrrole-2,5-dione were compared to the original reference [62,63], whereas the re-docking result of tolfenamic acid and COX-2 provided an RMSD value of 0.38 Å, while the re-docking result of ethyl 4-[(4-methylpyridin-2-yl) amino] piperidine-1-carboxylate at the iNOS binding site showed an RMSD value of 1.86 Å.

#### 4.4.4. Molecular Docking of Candidate Ligands

Molecular docking between the candidate ligands and target proteins was performed using AutoDock 4.2.6 software with default parameters. The grid boxes were set based on the native inhibitor with the number of points in the XYZ-dimension of 40 × 54 × 40; 60 × 40 × 70; 40 × 40 × 40; and 40 × 40 × 40, spacing 0.375 Å, and a center grid box at 6.693 × 23.248 × 58.264 (xyz); 10.113 × 4.042 × 13.511 (xyz); 165.756 × 186.098 × 192.976 (xyz); and 55.022 × 21.817 × 78.677 (xyz) for NF-κB, AP-1, COX-2, and iNOS, respectively. The conformation with the lowest binding energy was selected to evaluate the protein–ligand interaction using BIOVIA Discovery Studio 2020.

### 4.5. Lipinski’s Rule and Pharmacokinetic Property Analysis

To predict the drug-like properties of the identified compounds, physicochemical descriptors were computed by the SwissADME online database (http://www.swissadme.ch (accessed on 10 December 2022)) [67]. Regarding the Lipinski’s rule of five parameters, compounds that had molecular weight ≤ 500; a number of hydrogen bond acceptors ≤ 10; a number of hydrogen bond acceptors ≤ 5; and MlogP ≤ 4.15 were considered to be drug-like compounds [23]. In addition, the pharmacokinetic properties of the small molecules were predicted using SwissADME and preADMET (https://preadmet.qsarhub.com/toxicity (accessed on 10 December 2022)) online servers.

### 4.6. Cell Culture

The immortalized BV-2 mouse microglial cell line (Cat. #ABC-TC212S) was obtained from AcceGen Biotech (Fairfield, NJ, USA). This cell line was cultured in DMEM supplemented with 10% FBS, 100 U/mL penicillin, and 100 mg/mL streptomycin in a humidified incubator with 5% CO2 at 37 °C. The cells were grown to reach approximately 80% confluency before starting experiments. To evaluate the inflammatory responses in BV-2 cells, TNF-𝛼 at 10 ng/mL was used as an inflammatory inducer in the presence or absence of selected concentrations of CNSE for the indicated time.

### 4.7. Cell Viability Assay

To determine the viability of BV-2 cells, a 3-(4,5-Dimethylthiazol-2-yl)-2, 5-Diphenyltetrazolium Bromide Tetrazolium (MTT) assay was performed by detecting metabolic activity in the cells. Briefly, cells were seeded on a 96-well culture plate and incubated for 24 h. Next, the different doses of CNSE were treated with or without 10 ng/mL of TNF-𝛼 for 24 h. After the exposure time, 5 μg/mL of MTT solution was added for 4 h, the purple formazan products were dissolved in DMSO, and the absorbance was read at 570 nm using an EnSpire^®^ Multimode Plate Reader (Perkin-Elmer, Waltham, MA, USA). Data are shown as a percentage relative to control cells (untreated group).

### 4.8. Cell Morphological Analysis

To visualize the morphology of cells, cells were plated on a six-well culture plate and treated with the extract in combination with TNF-𝛼 for 24 h. Then, the cells were captured at 10× magnification using a Zeiss Model Axio Observer A1 phase-contrast microscope (Carl Zeiss, Jena, Germany). The results were analyzed by ImageJ software version 1.53k and expressed as a proportional percentage of round cells and spindle cells.

### 4.9. Quantitative Real-Time PCR Analysis

After treatment of cells with CNSE in the presence or absence of TNF-𝛼, total RNA was extracted using a Trizol reagent according to the manufacturer’s protocol. Then, 1 μg/mL of RNA was converted to cDNA using an AccuPower RT-premix. qPCR was performed using cDNA as templates for gene expression analysis under the following steps: predenaturation at 95 °C for 10 min, denaturation at 95 °C for 20 s, annealing at 58 °C for 40 s, and melting curve analysis. All primer sequences are as follows: IL-1β (forward 5′-GAAATGCCACCTTTTGACAGTG-3′; reverse 5′-CTGGATGCTCTCATCAGGACA-3′), TNF-𝛼 (forward 5′-GATCGGTCCCCAAAGGGATG-3′; reverse 5′-TAGCAAATCGGCTGACGGTG-3′), IL-6 (forward 5′-TCTTGGGACTGATGCTGGTG-3′; reverse 5′-CAGGTCTGTTGGGAGTGGTA-3′), and β-actin (forward 5′-GGCTGTATTCCCCTCCATCG-3′; reverse 5′-CCAGTTGGTAACAATGCCATGT-3′). The relative expression of specific genes was normalized with the internal control, which is β-actin, and calculated using the 2^−ΔΔCt^ method.

### 4.10. Immunoblotting Analysis

BV-2 cells were seeded on a six-well culture plate and subsequently pretreated with extracts with or without TNF-𝛼. The total protein was isolated using a cold 1X RIPA buffer containing proteinase inhibitor and phenylmethylsulfonyl fluoride (PMSF) and then centrifuged at 12,000 rpm for 10 min at 4 °C. The cell lysates were collected and the concentrations were determined by Bradford reagent with bovine serum albumin (BSA) as a standard protein. An equal concentration of protein for each sample was loaded to 12% (*v*/*v*) sodium dodecyl sulfate-polyacrylamide gel and subsequently transferred to a polyvinylidene difluoride (PVDF) membrane. Then, the membranes were blocked for 1 h with 5% skim milk in Tris-buffered saline, 0.1% Tween 20 (TBS-T), followed by incubation with antibodies specific for p-p65 (1:1000), p65 (1:1000), p-IκB-α (1:1000), IκB-α (1:1000), p-ERK1/2 (1:1000), ERK1/2 (1:1000), p-p38 (1:1000), p38 (1:1000), HO-1 (1:1000), and β-actin (1:1000), at 4 °C overnight. The β-actin was determined in a separated gel of protein, which was used as the internal control. At the end of the incubation period, all membranes were washed with TBS-T and incubated with horseradish peroxidase (HRP)-conjugated secondary antibody (1:8000) for 45 min at room temperature. Specific protein bands were captured using an Amersham^TM^ 800 Image Quant 800 (Cytiva, Marlborough, MA, USA) with an enhanced chemiluminescence (CL) reagent and then quantified using ImageJ software.

### 4.11. Enzyme-Linked Immunosorbent Assay (ELISA)

Cells were plated on a 6-well culture plate and preincubated with CNSE for 24 h, followed by TNF-𝛼 for another 24 h. The cell supernatant was retrieved, and cell debris was removed by centrifugation. The levels of TNF-𝛼 and IL-6 cytokines were measured using the commercial kit TNF-𝛼 and IL-6 ELISA following the manufacturer’s instruction.

### 4.12. Dual-Luciferase Assay

The pNF-κB-Luc and pRL-null plasmids were transfected in BV-2 cells using a PolyJetTM in vitro DNA transfection reagent (SignaGen, Frederick County, MD, USA). Then, CNSE, followed by TNF-𝛼, was added to transfected cells and the luciferase reporter assay was carried out by measuring the relative luciferase activity using a SynergyTM HTX multi-mode microplate reader (Biotek Instruments, Winooski, VT, USA).

### 4.13. Statistical Analysis

All experiments were carried out at least in triplicate and the data are expressed as the means ± standard variation. The statistical significance was considered as a *p*-value less than 0.05 by performing a one-way ANOVA with Tukey’s multiple comparison analysis with the SPSS statistics version 19 software.

## 5. Conclusions

**Figure 10:**
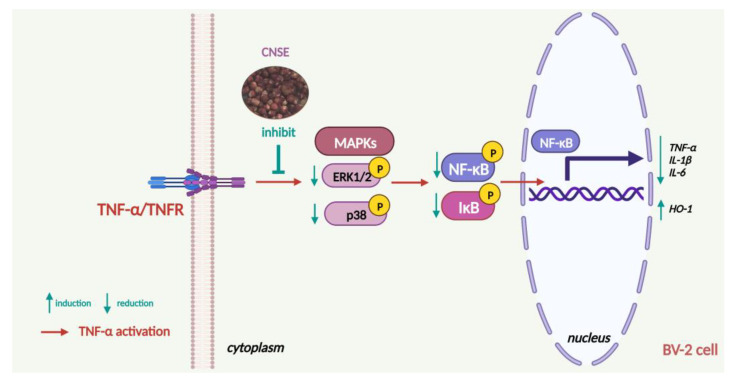
The proposed schematic overview of the effects and molecular mechanism of CNSE on protecting TNF-α-induced neuroinflammation in BV-2 mouse microglial cells (created by BioRender.com).

In our current study, CNSE exhibits antineuroinflammatory properties in BV-2 mouse microglial cells in response to TNF-α (Figure 10). CNSE decreases the expression of proinflammatory cytokines, including TNF-α, IL-1β, and IL-6, at both the gene and protein levels. These suppressive effects can be regulated through MAPKs and the NF-κB signaling pathway due to a decrease in the protein levels of ERK, p38MAPK, p65, and IκB-α. Moreover, CNSE treatment could also promote the expression of HO-1 protein in BV-2 cells. The bioactive phytochemicals in CNSE are identified, especially alpinetin and aurentiacin. They exerted possible inhibitory effects on NF-κB and both iNOS and COX-2 enzymes. However, CNSE and its identified compounds need to be further investigated for their antineuroinflammatory effects and involved molecular pathways in other glial cells, including primary cells, animal models, and clinical trials. Therefore, our findings indicate a helpful potential of CNSE for the prevention or treatment of neuroinflammatory disorders associated with neuroglial cells, particularly microglial cells.

## Figures and Tables

**Table 1:** Molecular docking results of top 5 identified compounds to NF-κB binding site.

Ligand	Binding Energy (kcal/mol)	Inhibition Constant (μM)	Amino Acid Interaction		
			Hydrogen Bond	Hydrophobic Bond	Other
3,5-dimethyl-4-[(2-nitrophenyl)diazenyl]pyrazole-1-carbothioamide (native inhibitor)	−6.33	22.78	ARG246 (3) GLN247	LYS218 (2) VAL248 ARG246 (2) ALA192	-
Ellagic acid	−7.31	4.41	ARG33 (2) LYS218 (2) ARG187	LYS218 (2) ARG187 (2) ALA192	-
Alpinetin	−6.17	30.19	ARG33 (2) ASN186 GLU193 ASP217	ALA192 ARG187	-
Aurentiacin	−6.01	39.06	ASN186 (2) LYS218 (2) ARG246 GLU193	ALA192 (2) LYS218 ARG187	ASP217
Brassitin	−5.11	178.17	ARG33 (2) ASP217	ARG33 ALA192	-
Ferulic acid	−4.52	488.99	ASN186 LYS218 (2) ASP217 ARG187	ALA192 (2) LYS218	ASN186

**Table 2:** Molecular docking results of top 5 identified compounds to AP-1 binding site.

Ligand	Binding Energy (kcal/mol)	Inhibition Constant (μM)	Amino Acid Interaction		
			Hydrogen Bond	Hydrophobic Bond	Other
1-[[6-methoxy-2-(2-thienyl)quinazolin-4-yl]amino]-3-methyl-pyrrole-2,5-dione (native inhibitor)	−9.29	0.016	SER16 ARG17 LYS20 (2) DG208 DA209 (3)	ALA13 ARG17 (4) LYS20 (3) LEU21 (2) DG208 (4)	ARG17 LYS20 DA209 (2)
Aurentiacin	−7.69	2.32	LYS20 DG208	ARG17 (3) LEU21 (2) LYS20 (2) DG208 DA209	ARG17 LYS20 DA209
Alpinetin	−7.22	5.06	ARG17 DG208	ARG17 (2) LYS20 LEU21 DG208	ARG17 LYS20
Ellagic acid	−7.06	6.67	ARG17 (2) LYS20 DG208 (2) DA209 (3) DC210	ALA192 (2) ARG17 (4) LYS20 (2)	ARG17 (2) LYS20
Ferulic acid	−6.72	11.81	ARG17 (2) LYS20 DA209	ARG17 (2) LYS20 LEU21	LYS20
Brassitin	−6.7	12.26	SER16 LYS20 DG208	ALA13 ARG17 (3) LYS20 LEU21 DT207 DG208 (2)	LYS20 (2) DG208 (3)

**Table 3:** Molecular docking results of top 5 identified compounds to iNOS binding site.

Ligand	Binding Energy (kcal/mol)	Inhibition Constant (μM)	Amino Acid Interaction		
			Hydrogen Bond	Hydrophobic Bond	Other
Ethyl 4-[(4-methylpyridin-2-yl) amino] piperidine-1-carboxylate (native inhibitor)	−6.91	8.64	TYR347 VAL352	TRP346 (2) TYR347 PRO350 (2) VAL352 ASN370	-
Ellagic acid	−6.32	23.46	ILE265 (2) ARG266 (2) ALA351 TYR373 GLU377 ARG388	GLN263	ASP382 (2)
Aurentiacin	−6.13	32.37	TYR347 ALA351 TYR373 GLU377 (2)	TRP346 (2) PRO350 VAL352 TYR373 ARG381	-
Alpinetin	−6.11	33.02	VAL352 TRP372	PRO350 (2) PHE369	-
Brassitin	−5.63	75.29	TYR347 ALA351 GLU377	PRO350 (2) VAL352 TYR373 (2)	TYR347 TYR373
Ferulic acid	−4.71	323.29	ARG199 CYS200 GLY202 PRO350	PRO350 VAL352	CYS200

**Table 4:** Molecular docking results of top 5 identified compounds to COX-2 binding site.

Ligand	Binding Energy (kcal/mol)	Inhibition Constant (μM)	Amino Acid Interaction		
			Hydrogen Bond	Hydrophobic Bond	Other
Tolfenamic acid (native inhibitor)	−8.13	1.1	TYR385 SER530	VAL116 VAL349 (2) LEU352 VAL523 ALA527 (4) LEU531 (2)	-
Alpinetin	−8.14	1.08	TYR355 SER530	VAL349 (2) LEU352 LEU359 PHE518 GLY526 ALA527 (2) LEU531	MET522
Aurentiacin	−8.03	1.3	ARG120 TYR355	MET113 VAL116 (2) VAL349 (3) LEU352 TYR355 LEU359 TRP387 GLY526 ALA527 (3) LEU531 (2)	MET522
Ellagic acid	−6.89	8.94	TYR355 SER530	VAL349 (2) LEU352 (4) VAL523 (2) GLY526 (3) ALA527 (6)	-
Brassitin	−6.29	24.61	SER530	PHE205 TYR348 LEU352 (2) TYR385 PHE518 VAL523 (2) GLY526 (2) ALA527 (3)	TYR348 TYR385 MET522
Ferulic acid	−5.07	193.72	TYR355 SER353	LEU352 PHE381 TYR385 MET522 VAL523 (2) GLY526 ALA527 (2)	MET522

**Table 5:** Prediction of drug-likeness of candidate ligands by Lipinski’s rule of five parameters.

Compound	Molecular Weight (≤500)	#H-Bond Acceptors (≤10)	#H-Bond Donors (≤5)	MLOGP (≤4.15)	Lipinski #Violations (≤1)
Alpinetin	194.18	4	2	1	0
Aurentiacin	298.33	4	1	2.31	0
Brassitin	302.19	8	4	0.14	0
Ellagic acid	220.29	1	2	1.4	0
Ferulic acid	270.28	4	1	1.52	0

**Table 6:** ADMET-predicted properties of possible ligands in CNSE.

Pharmacokinetic Property	Alpinetin	Aurentiacin	Brassitin	Ellagic Acid	Ferulic Acid
GI absorption	High	High	High	High	High
Pgp substrate	Yes	No	No	No	No
log Kp (skin permeation) (cm/s)	−6.07	−5.14	−6.22	−7.36	−6.41
BBB permeant	Yes	Yes	Yes	No	Yes
CYP1A2 inhibitor	Yes	Yes	Yes	Yes	No
CYP2C19 inhibitor	Yes	Yes	Yes	No	No
CYP2C9 inhibitor	No	Yes	No	No	No
CYP2D6 inhibitor	No	Yes	No	No	No
CYP3A4 inhibitor	Yes	Yes	No	No	No
Carcinogenicity (mouse)	Negative	Negative	Negative	Negative	Negative
hERG inhibition	Medium risk	Medium risk	Medium risk	Low risk	Medium risk

## Data Availability

Data available on request from the authors.

## Supplementary Materials

The following supporting information can be downloaded at: https://www.mdpi.com/article/10.3390/molecules28073057/s1, Figure S1: Original photographs of the full-length blots at three independent experiments of each protein marker of Figure 8A; Figure S2: Original photographs of the full-length blots at three independent experiments of HO-1 protein of Figure 9A.

## Sample Availability

Samples of the compounds are not available from the authors.
