# Supplementary material for: Cleistocalyx nervosum var. paniala Berry Seed Protects against TNF-α-Stimulated Neuroinflammation by Inducing HO-1 and Suppressing NF-κB Mechanism in BV-2 Microglial Cells
Source: Molecules. 2023 Mar 29;28(7):3057. doi: 10.3390/molecules28073057 (PMC10095692; doi:10.3390/molecules28073057)

Original Images for Blots/Gels

Supplementary Figure S1. Original photographs of the full-length blots at three independent experiments of each protein marker of Figure 8A.

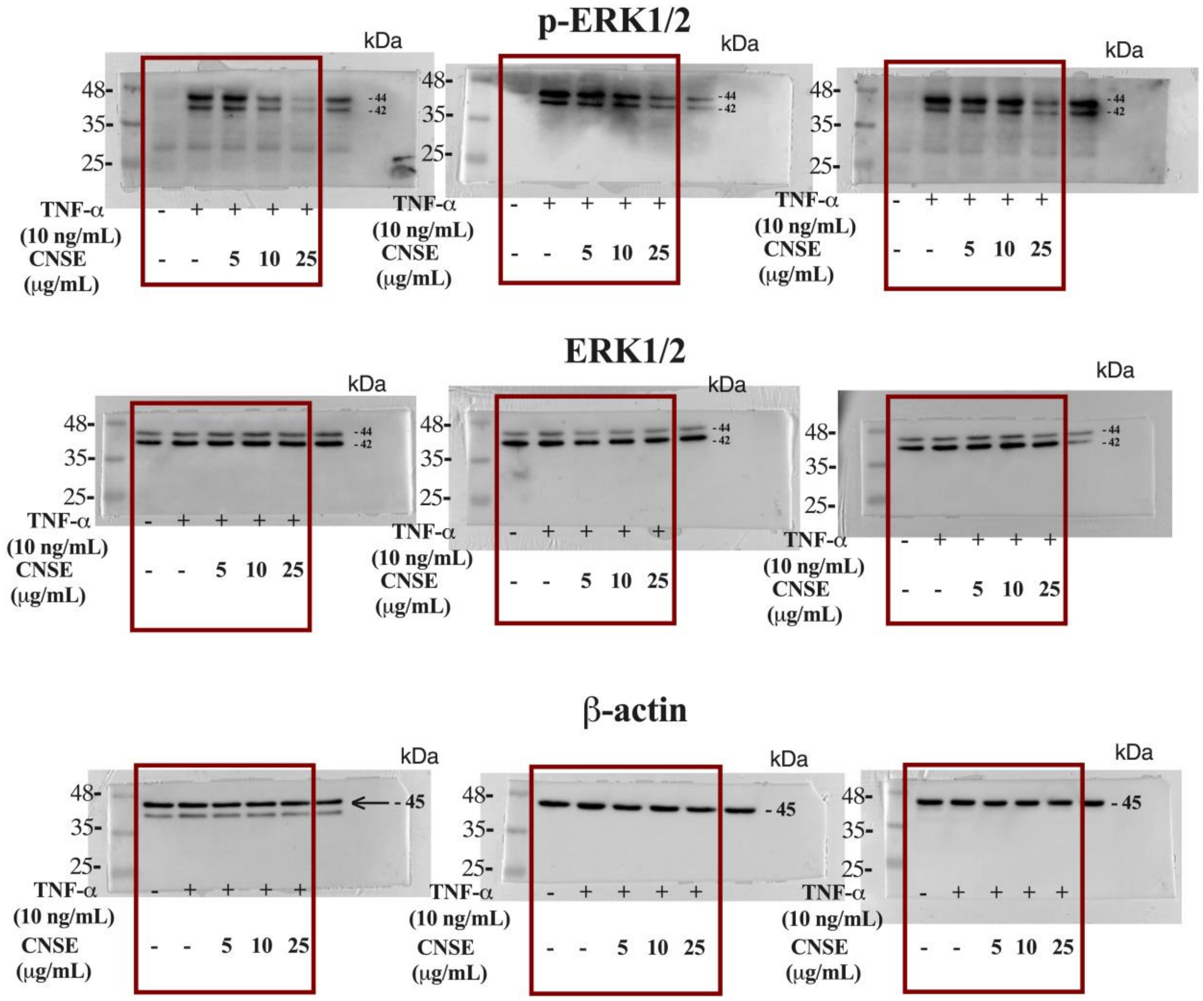

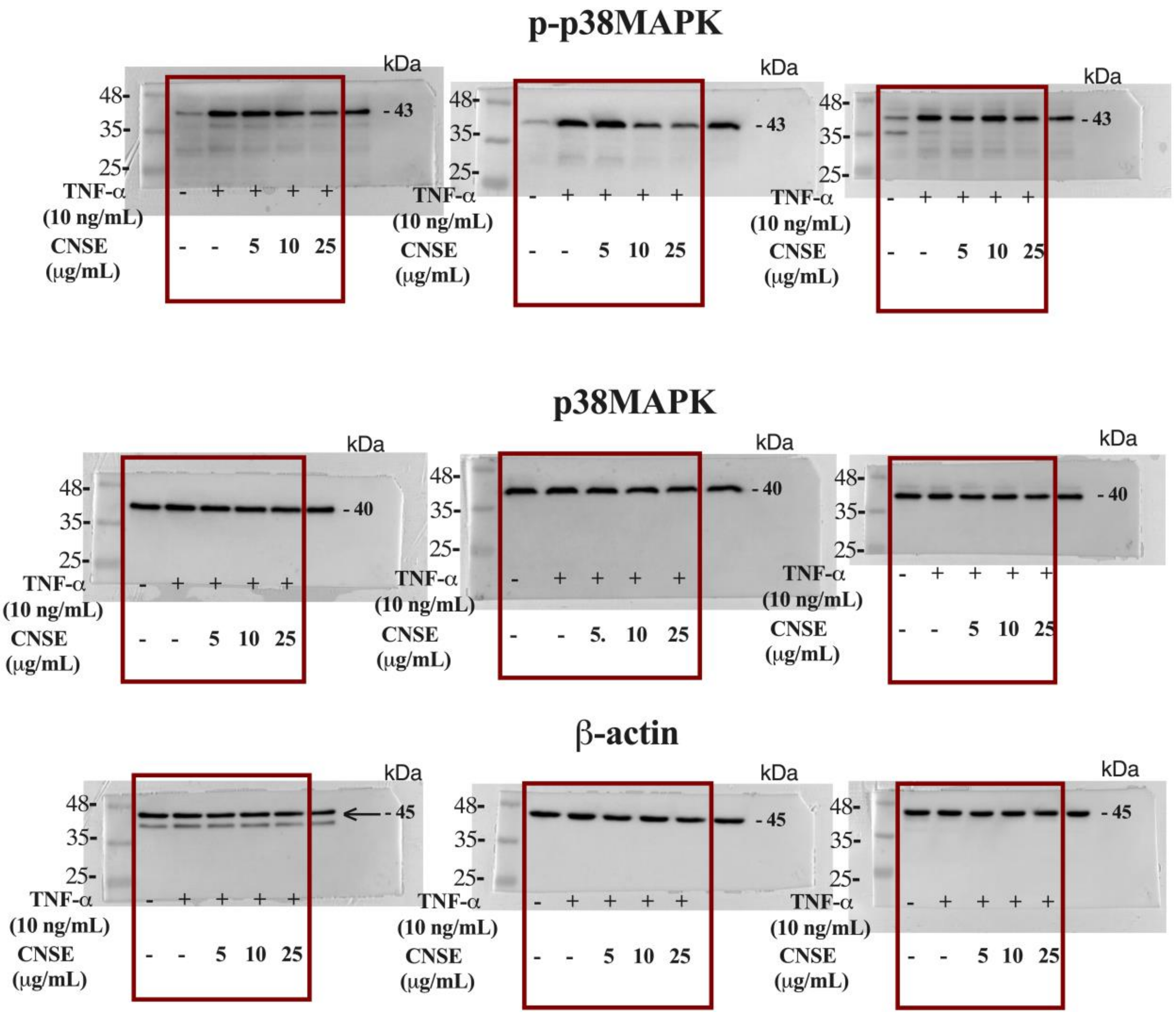
p-p38MAPK
kDa
48-
35-
25-
- 43
TNF-α (10 ng/mL) - + + + +
CNSE (μg/mL) - - 5 10 25
p38MAPK
- 40
TNF-α (10 ng/mL) - + + + +
CNSE (μg/mL) - - 5 10 25
β-actin
- 45
TNF-α (10 ng/mL) - + + + +
CNSE (μg/mL) - - 5 10 25

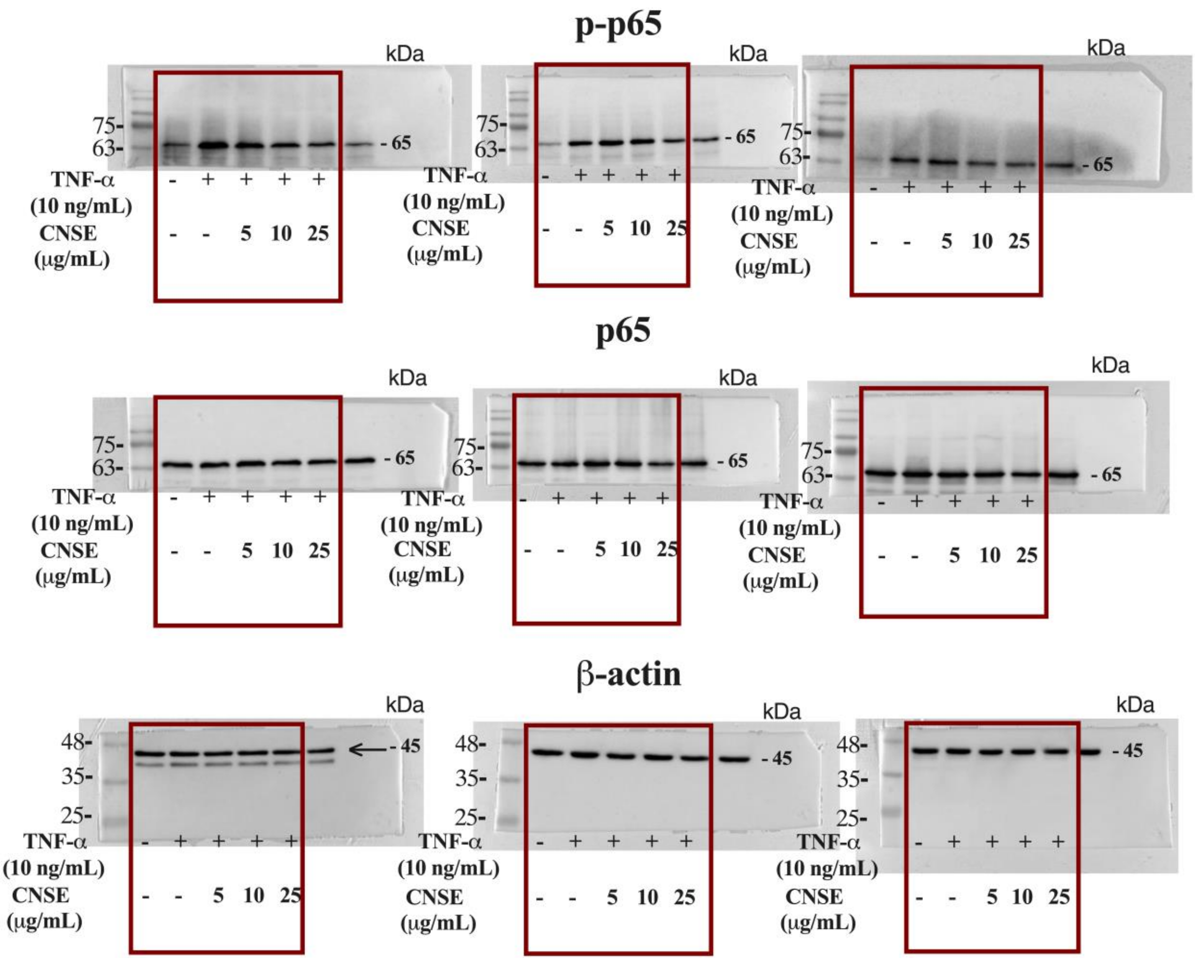
p-p65
p65
β-actin
kDa
75-
63-
- 65
48-
35-
25-
- 45
TNF-α (10 ng/mL)
- + + + +
CNSE (μg/mL)
- - 5 10 25

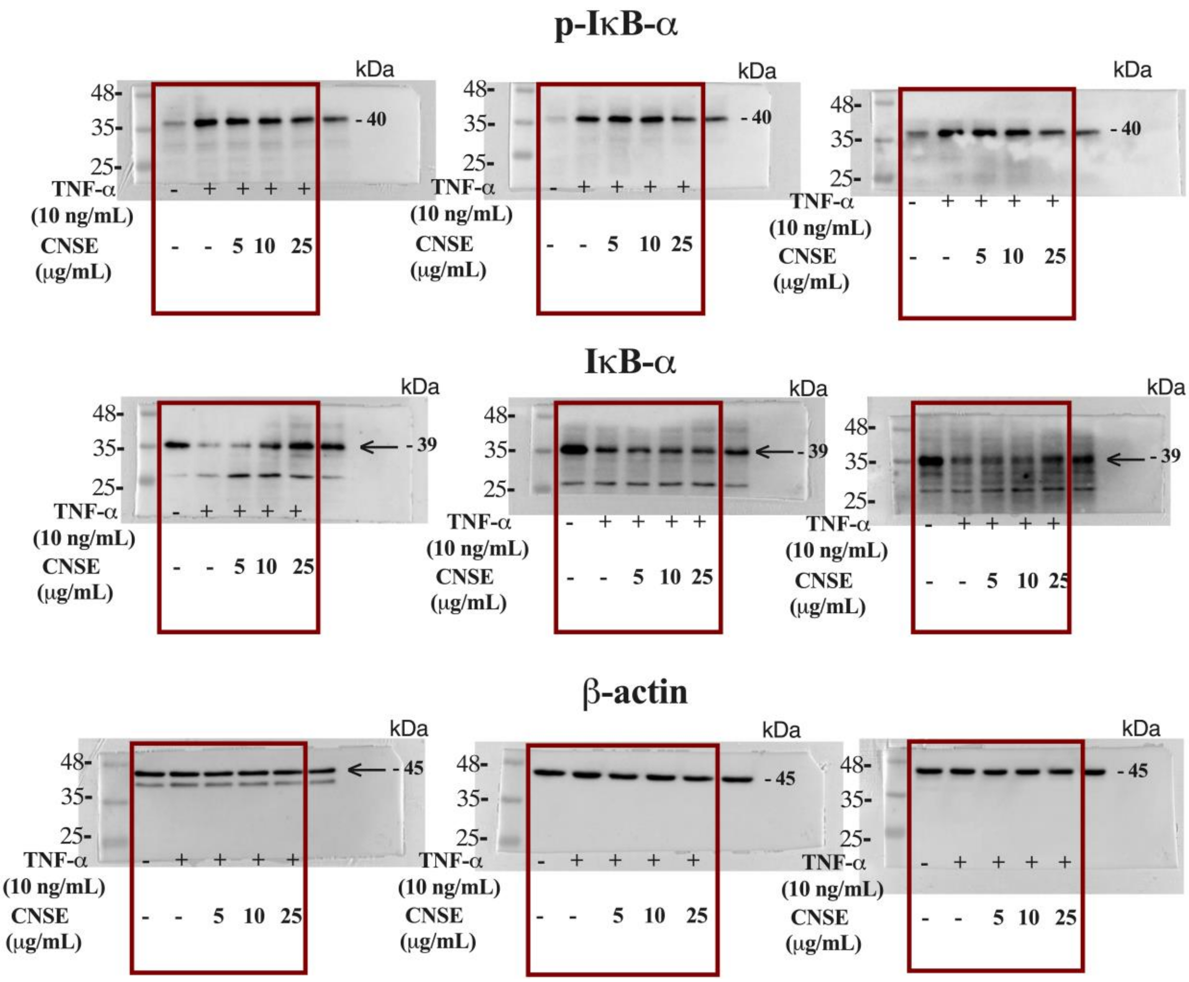

p-IκB-α
kDa
48-
35-
25-
- 40
TNF-α
(10 ng/mL)
- + + + +
CNSE
(μg/mL)
- - 5 10 25
IκB-α
- 39
β-actin
- 45

Supplementary Figure S2. Original photographs of the full-length blots at three independent experiments of HO-1 protein of Figure 9A.

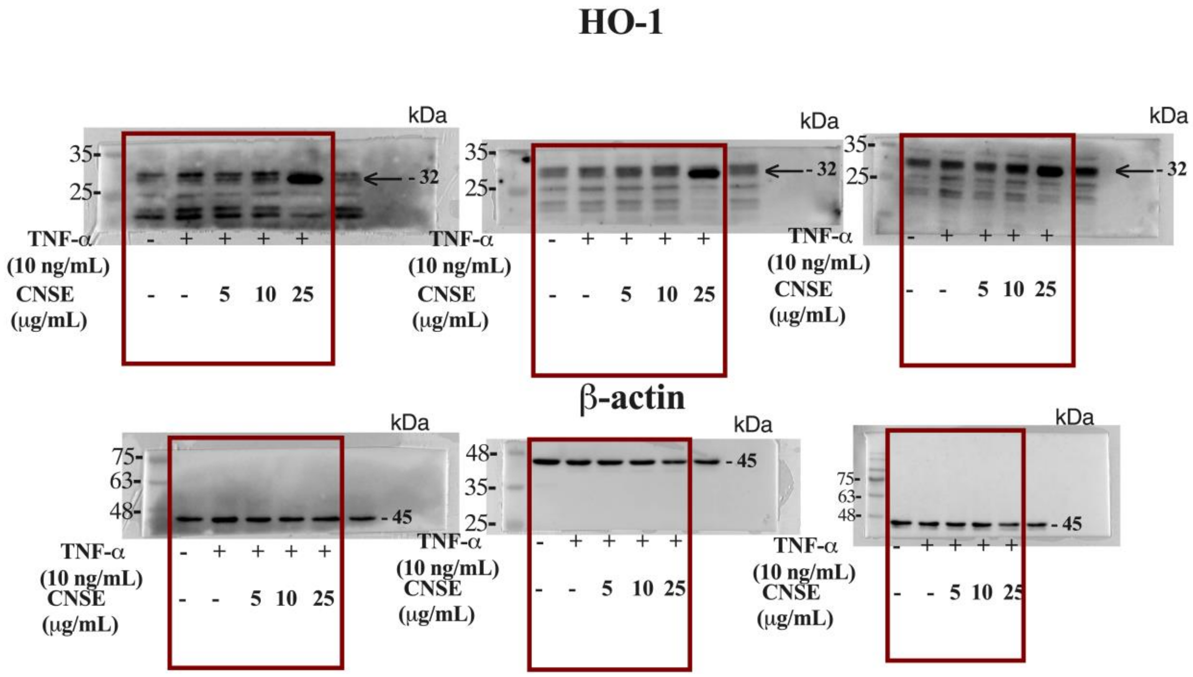

Supplement: Supplementary file 1 [file molecules-28-03057-s001.zip › molecules-2287930-supplementary.pdf]
